# Highly Selective and Reproducible Electrochemical Sensing of Ascorbic Acid Through a Conductive Polymer Coated Electrode

**DOI:** 10.3390/polym11081346

**Published:** 2019-08-13

**Authors:** Salma Bilal, Ayesha Akbar, Anwar-ul-Haq Ali Shah

**Affiliations:** 1 National Centre of Excellence in Physical Chemistry, University of Peshawar, Peshawar 25120, Pakistan; 2 TU Braunschweig Institute of Energy and Process Systems Engineering,Franz-Liszt-Straße 35, 38106 Braunschweig, Germany; 3 Institute of Chemical Sciences, University of Peshawar, Peshawar 25120, Pakistan

**Keywords:** polyaniline, ascorbic acid sensing, electrochemical polymerization, dodecylbenzene sulphonic acid

## Abstract

The surface of an Au-disc electrode was modified through electro polymerization of aniline, in the presence of dodecyl benzene sulphonic acid (DBSA) and sulphuric acid (H_2_SO_4_) solution. The polymerization conditions were pre-optimized so that micelle formation and solution coagulation could be minimized and surfactant doped polyaniline film could be obtained through a quick, simple and one step polymerization route. The synthesized material was characterized via Fourier transform infrared (FTIR) spectroscopy, thermogravimetric analysis (TGA), scanning electron microscopy (SEM) and cyclic voltammetry (CV). The effective surface area of the Au-disc, calculated through cyclic voltammetry, was immensely increased through a polyaniline (PANI) coating (0.04 and 0.11 cm^2^ for bare and PANI coated gold respectively). The modified electrode was utilized for ascorbic acid (AA) sensing. The changing pH of electrolyte and scan rate influenced the PANI electrode response towards AA. The modified electrode was highly selective towards AA oxidation and showed a very low limit of detection i.e. 0.0267 μmol·L^–1^. Moreover, the PANI coating greatly reduced the sensing potential for AA by a value of around 140 mV when compared to that on a bare gold electrode.

## 1. Introduction

Chemical sensors are of vital importance in different fields including monitoring of different species in the atmosphere, in industrial processes, biomedical analysis, and food quality control [1]. Among many analytes, ascorbic acid (AA), which is an important biomolecule, is frequently detected as it is a main nutrient and antioxidant found in vegetables, fruits and in other naturally occurring systems [2]. It is useful in activating an immune response, and it contributes in wound healing, iron absorption, osteogenesis collagen formation, and preservation of capillaries, bones and teeth [3]. It is usually recommended in the treatment of certain diseases such as Alzheimer’s, infertility HIV infections, atherosclerosis and cancer [4]. The normal level of AA in human plasma is in between 50 to 70 µM [5]. 

A high level of AA can cause gastric irritation, and its metabolites can lead to renal issues, while the deficiency of AA is responsible for the illness scurvy [6]. AA determination is also essential in the agricultural field. Some natural processes in food may get disturbed due to excessive amounts of AA which is responsible for deterioration in the taste and aroma [7]. 

Various techniques such as chromatography [8], chemiluminescence [9], and spectrometry [10] have been reported for the detection of AA. However, these techniques are expensive and require highly skilled personnel working on complex equipment. These techniques also involve difficult analytical procedures in multi sample preparation. Therefore, extensive research is underway develop a simple, fast, reliable and cost effective technique adequate for in-situ and on-time analyte detection [11]. 

In this context, electrochemical methods are more suitable, reliable, economic and user-friendly in-field applications. The electrochemical techniques have linear wide range responses with exceptional repeatability, accuracy and low detection limits [12]. During electrochemical sensing, electrodes are used for passing current to solution and the resulting electrical signals, caused by the electrochemical reactions occurring between analyte and electrode, are recorded.

The instrumentational setup of an electrochemical technique is mostly compact, portable and simple due to electrode miniaturization which makes this technique more effective for sensing analytes [13]. Generally, a three electrode setup including reference, counter and working electrodes is employed in electrochemical techniques. However, it has been reported that the direct oxidation of AA is irreversible at bare electrodes (such as glassy carbon, screen printed and gold). High over potential is required which results in low selectivity, fouling of electrodes, and poor reproducibility. Therefore, surface modification of electrodes leading to selective and sensitive AA detection with less over potential has received much attention [14]. Different materials have been employed for surface modification of the working electrode including silicon, metal oxides, metals, conducting polymers and carbon nanotubes [15].

Conducting polymers, have been particularly investigated because of their simple fabrication method, biocompatibility, optical and characteristic physical properties. Conducting polymer based sensors offer attractive advantages. They are effective at room temperature, diverse in structure and easy to fabricate [16,17,18]. The conducting polymer polyaniline (PANI) has exceptional properties, including stable mechanisms of electrical conduction, reversible and simple doping–dedoping ability, easy synthesis, high environmental stability and low cost. These properties make it a potential candidate for applications in electrochemical sensors [19,20,21]. It is such a versatile material that its properties can be very easily tuned, by developing different synthetic strategies, to make it suitable for various applications [22].

Most of the promising properties of PANI are observable when it is in its doped and conducting state. PANI can be doped simultaneously with many inorganic and organic materials in order to change its structure, rise mechanical strength, increase conductivity, improve solubility and to create more active sites to convert the targeted molecule into easily detectable species [22,23]. In previous reports electrodes modified with PANI in its doped state were effectively used for the electro-catalytic detection of AA [24,25]. It is supposed that PANI will not only have a large and adhesive surface area, but also provide a conductive medium which will favorably support the dopant materials for their effective role in AA electro-oxidation. These works show that PANI electrodes have a great potential to sense AA. 

We report here a very simple and reproducible way of electrode modification by electrooxidation of aniline in the presence of dodecylbenzene sulfonic acid (DBSA) and sulphuric acid (H_2_SO_4_) solution. Although surfactant molecules such as DBSA can impart very promising properties to PANI, the polymerization of aniline in the presence of surfactants always remained a challenge due to micellization and coagulation. Unlike the surface modification of electrodes with chemically synthesized and doped PANI, which often requires more than a week to synthesize the polymer to use it as coating for electrode surfaces, the present methodology is very simple and can be done in a single step in a very short time [26,27]. Different characterization techniques support formation of PANI containing both DBSA and sulfuric acid as dopants. This modified PANI electrode exhibits rapid electro-catalytic activity towards AA oxidation along with enhanced linearity, comparable low detection limit (LOD), high sensitivity and good selectivity.

## 2. Experimental

### 2.1. Chemicals

Sulphuric acid solution (97.5%), dipotassium hydrogen phosphate (K_2_HPO_4_) and potassium hydroxide (KOH) (Scharlau, Sentmenat, Spain ), potassium dihydrogen phosphate (KH_2_PO_4_) and dopamine (DA) (Merck, Kenilworth, NJ, USA), ascorbic acid (AA) (Merck, Kenilworth, NJ, USA), uric acid (UA) (Alfa Aesar, ), DBSA (Sigma Aldrich, Hamburg, Germany) were used as received. Deionized water (H_2_O) (Millipore, Burlington, MA, USA) was used for solution preparation. Aniline () was double distilled ( Merck, Kenilworth, NJ, USA) and stored in a refrigerator.

### 2.2. Instrumentation

Fourier-transform infrared spectroscopy (FTIR) analysis of PANI was carried out in the range of 400–4000 cm^−1^ by using IRAffinity-1S Shimadzu Spectrophotometer (Shimadzu, Tokyo, Japan). Thermal stability of PANI samples were determined by using Perkin Elmer, Diamond series (Waltham, MA, USA) at heating rate of 10 °C/min under a nitrogen atmosphere. Surface imaging of the synthesized samples was carried out with a scanning electron microscope (SEM) (Helios G4 CX DualBeam microscope equipped with OctaneElite, EFI, Berlin Germany). Electrochemical characterization was done in a three electrode cell using Reference 600 ZRA potentiostat (Gamry, Warminster, PA, USA). A gold disc, saturated calomel electrode (SCE) and gold sheet were utilized as working, reference and counter electrode, respectively. 

### 2.3. Cleaning of the Au-disc Electrode

The Au-disc electrode was first mechanically cleaned by polishing with alumina for 5 min. This polished Au-disc electrode was then washed with distilled water and further electrochemically cleaned by applying potential cycling in the range of −0.2 to 1.45 V at a scan rate of 100 mV/s in 0.5 M sulfuric acid solution, until the cyclic voltammograms become stable (40 cycles). After this, its cyclic voltammogram was recorded in 4 mM K_3_[Fe(CN)_6_]/1 M KNO_3._ The ΔE_p_ of the bare Au- electrode was found to be 90 mV, indicating less surface imperfection of the electrode [28].

### 2.4. Fabrication of the Modified PANI Electrode 

**Scheme 1:**
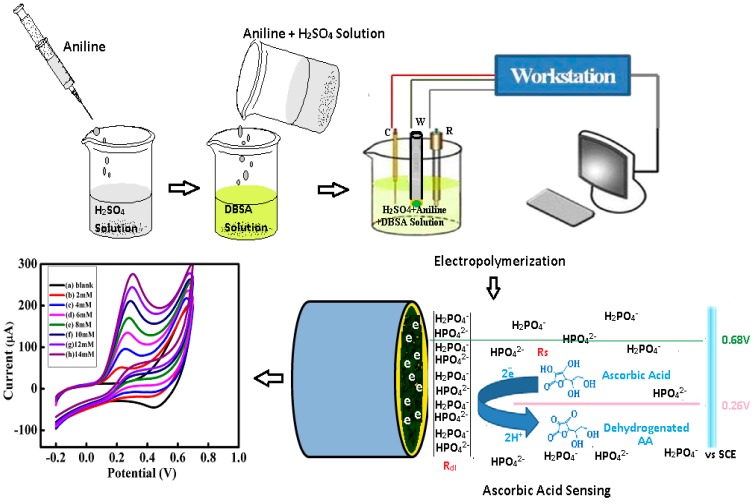
Schematic representation of polyaniline (PANI) electrode preparation and electrochemical oxidation of ascorbic acid (AA). Dodecylbenzene sulfonic acid (DBSA).

The surface of Au-disc electrode was modified with PANI by electro-oxidation of aniline in a single cell consisting of three electrodes (Scheme 1). 0.05M aniline was added to 1M H_2_SO_4_ solution, sonicated for 10 to 15 minutes and was then poured into 0.025M DBSA solution. This mixture was thoroughly shacked and transferred to the electrochemical cell where it was subjected to 10 potential cycles in the range of −0.2 to 0.8 V at 20 mV/s using Au-disc working electrode. A dark green layer was observed on the surface of the electrode. The modified electrode after polymerization was washed with distilled H_2_O and then dried. The PANI modified Au-disc was used as the working electrode for AA oxidation. Chronoamperometry was conducted to monitor AA at an optimized 0.24 V.

### 2.5. Preparation of the AA Solutions

The AA solutions were prepared in phosphate buffer (PB). PB solutions (PBS) of varying pH were made by means of the Henderson–Hasselbalch equation [29]. After PBS preparation, AA (0.88 g) was added to 0.1M PBS in order to make 100 mM AA stock solution which was further utilized for the preparation of different concentrated AA solutions.

## 3. Results and Discussions

### 3.1. Electropolymerization of Aniline

**Figure 1:**
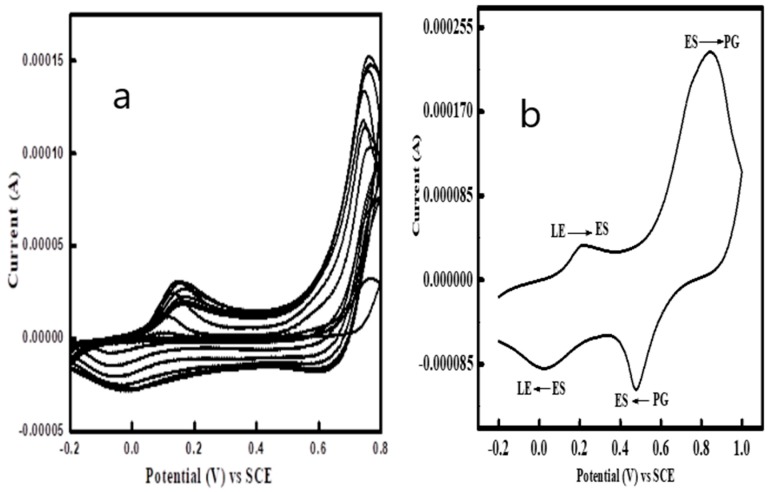
(**a**) Cyclic voltammograms recorded on Au-disc electrode at scan rate of 50 mV/s during polymerization of aniline from an acidic solution containing DBSA. (**b**) Cyclic voltammogram of the PANI film recorded in 1 M sulfuric acid at scan rate of 50 mV/s, indicating the interconversion of PANI film between its reduced leucoemeraldine (LE), semi-oxidized and conducting emeraldine salt (ES) and oxidized pernagraniline (PG) forms.

Aniline polymerization was carried out via cyclic voltammetry and its characteristic voltammogram are shown in Figure 1a. During polymerization no faradic process was observed until 0.6 V in the initial forward scan, and after this potential, the polymer growth and nucleation occurs up to 0.8 V. The cathodic peak Epc was observed at 0.06 V during the initial backward scan. During the first cycle, the increased in current between 0.60–0.75 V shows the anilinium cationic radicals’ formation. This peak current is further enhanced when potential is scanned again which indicates the coupling of cation radicals to form benzenoid and quinoid structures [30].

At the completion of the 3^rd^ cycle, two visible anodic and cathodic peaks were developed indicating the polyaniline (PANI) thin layer formation at the bare Au-disc electrode. A green color film was observed on the surface of electrode after 10 cycles. This film was washed with deionized water, dried and then transferred to a monomer free electrolyte where a cyclic voltammogram was recorded in order to characterize the electrochemical response of polymer film. The potential was scanned between −0.2 and 1 V at the scan rate of 50 mV/s. 

**Scheme 2:**
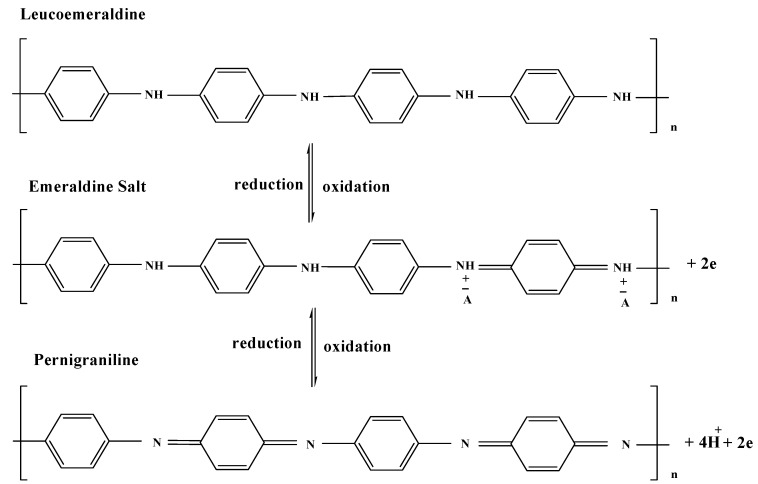
Interconversion of PANI into its different forms through oxidation reduction [32].

The interconversion of different PANIs between its different forms including reduced leucoemeraldine (LE), semi-oxidized and conducting emeraldine salt (ES) and oxidized pernagraniline (PG) [31] is indicated by the presence of two redox pairs in Figure 1b. The LE and ES inter conversion is shown by a redox pair at 0.12 and −0.002 V while the ES and PG interconversion is represented by a redox pair at 0.66 and 0.6 V. The interconversion of different PANI redox states is shown in Scheme 2. It is noteworthy to mention that the film obtained by electro-oxidation of aniline in the presence of DBSA in acidic solution showcases very well defined typical redox characteristics of PANI. Further insights into the properties of this PANI film were gained through different characterization techniques as discussed in the following sections. 

### 3.2. FTIR Analysis

**Figure 2:**
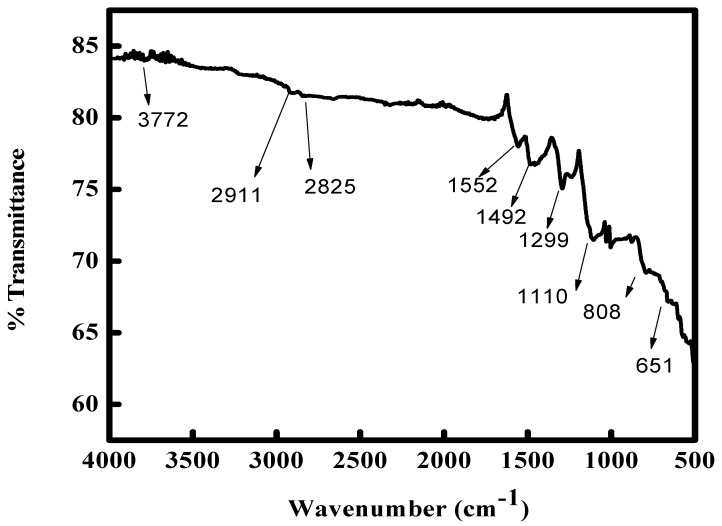
Fourier-transform infrared spectroscopy (FTIR) spectrum of the synthesized PANI.

The presence of different functional groups in the PANI back bone was confirmed by FTIR analysis (Figure 2). For this purpose the PANI film, washed with deionized water, was scratched from the electrode in its doped form, and dried in the form of powder. 

The benzenoid and quinoid stretching is denoted by the peaks at 1492 cm^−1^ and 1552 cm^−1^, respectively [33]. The polaron C–N* stretching is located at 1299 cm^−1^ indicating the conducting form of PANI [34]. The peak at 1110 cm^−1^ is attributed to the stretching of C–N= [35,36] while the peak at 808 cm^−1^ shows out of plane deformation of C–H [37]. The aliphatic C–H, due to DBSA, is represented by the bands at 2825–2911 cm^−1^. The HSO_4_¯ peak at 651 cm^−1^ proves the presence of acidic dopants in the PANI film [38]. The FTIR analysis effectively confirms the incorporation of both DBSA and sulfuric acid moieties into the PANI backbone.

### 3.3. SEM Analysis

**Figure 3:**
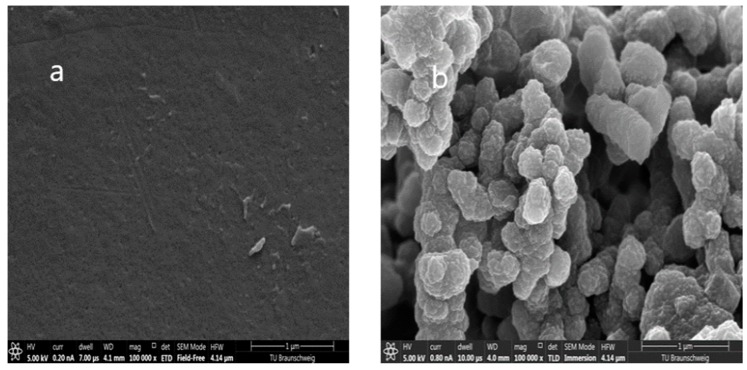
Scanning electron microscopy (SEM) image of (**a**) undoped PANI **(b**) PANI doped with DBSA and sulfuric acid.

The surface morphology of PANI in its undoped and doped state (Figure 3a,b respectively) was observed by taking scanning electron micrographs. Micro-spherical particles and compact globules can be seen in SEM images of doped PANI at high magnification. The surface morphology of PANI exhibits a cauliflower like structure. The DBSA presence in the polymer has a significant role in presenting this kind of morphology because it makes the PANI more granular, dense, and porous as compared to PANI without DBSA. This type of morphology is considered very important for application of PANI in sensors because it can offer more active sites to the target analyte leading to a low detection limit [39]. 

### 3.4. Thermal Analysis

**Figure 4:**
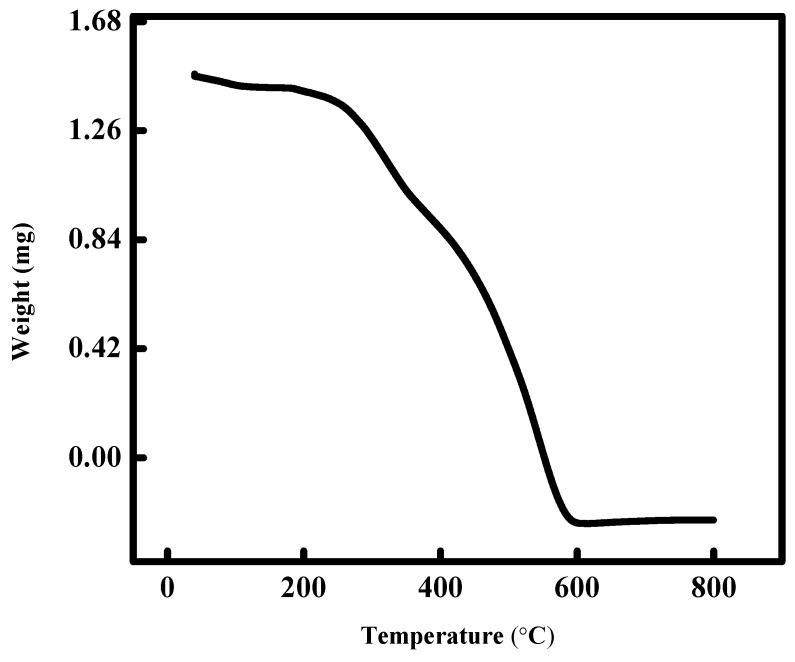
Thermogravimetric analysis (TGA) plot of the synthesized PANI.

The thermal decomposition and stability of PANI was determined via thermogravimetric analysis (TGA). The polymer mass vs temperature was measured between 40 to 800 °C. In Figure 4 the first weight loss at 248 °C indicates the removal of volatile materials i.e. adsorbed moisture on the sample surface, and residue of electrolyte and oligomers of lower molecular weights. The second weight loss at 250–450 °C is attributed to the removal of DBSA in the PANI. At 450–600 °C, the weight loss is due to thermo-oxidative PANI decomposition [40]. The TGA plot indicates that the synthesized PANI has quite high thermal stability. 

### 3.5. Effective Area of PANI Modified Electrode

The better sensing performance of PANI is often related to the increase in effective surface area of the working electrode when modified with PANI. To confirm this argument, effective surface area of the bare and PANI coated Au-disc electrode was determined as follows. 

**Figure 5:**
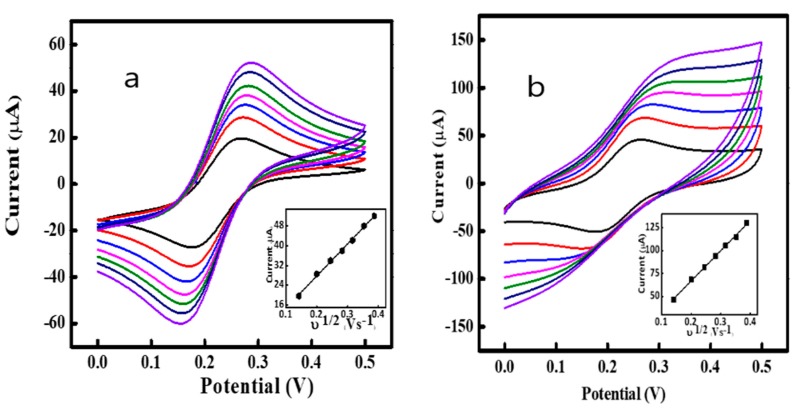
Cyclic voltammograms of (**a**) bare and (**b**) PANI modified Au disc electrodes, recorded in 4 mM K_3_Fe(CN)_6_ */*1 M KNO_3_ at different scan rates (20–150 mVs^−1^). Inset: Dependence of anodic peak current on square root of scan rate.

Cyclic voltammograms were recorded for bare and PANI modified electrodes in 4 mM K_3_[Fe(CN)_6_] in 1 M KNO_3_ (Figure 5a,b). The anodic peak potential E_pa_ of bare and modified PANI electrode was 0.266 and 0.258 V while the cathodic peak potential E_pc_ of bare and PANI modified electrode was 0.179 and 0.190 V. The E_pa_ and E_pc_ of bare as well as modified electrode shifted towards more positive and negative value, respectively when scan rate increases. Linear plot was obtained when anodic peak current *I_p_* was plotted vs square root of potential scan rate (Insets of Figure 5a,b). The Randles–Sevcik equation was used to measure the effective area of the bare and PANI coated electrodea [41].

*I*_p_ = (2.69 × 10^5^) *n*^3/2^ A υ^1/2^*D*^1/2^*C* (1)

where the current peak is denoted by *I*_p_, *n* represents the electron numbers involved during the reaction. Diffusion coefficient *“D”* of K_3_[Fe(CN)_6_] is approximately equal to 6.7 × 10^-6^ cm^2^/s, the K_3_[Fe(CN)_6_] molar concentration is *C* = 4 mM. The 0.04 and 0.11cm^2^/s are the calculated values of electroactive surface area of the bare and modified PANI electrode respectively. These values confirm that the PANI coating increases the active surface area of the Au disc electrode, hence providing more active sites for analyte oxidation [42]. 

### 3.6. Ascorbic Acid (AA) Sensing by PANI

**Scheme 3:**
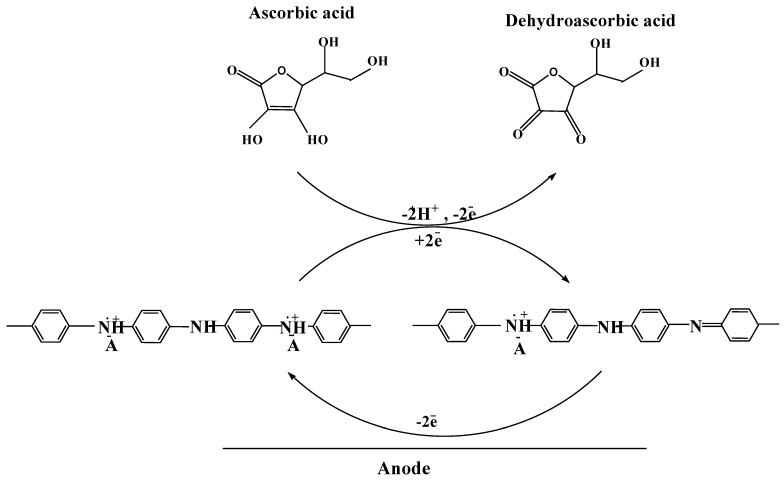
Sensing mechanism of AA by PANI. Charge neutrality is retained by dopant ions.

PANI present in the form of emeraldine salt, is more highly conductive than the emeraldine base state due to dopant ions existence in PANI. Here the DBSA has a significant role in the sensing mechanism because of its large size and presence of anionic sulphate (SO^3-^) group and a dodecyl (C_12_H_25_) group. The dodecyl (C_12_H_25_) group is responsible for non-polar interaction with the surrounding aqueous medium and it resists the PANI being phased out. The interaction between the chains of conjugated PANI is also reduced due to DBSA. Thus DBSA in PANI creates more potential sites for better sensing of AA. In the current situation when AA comes near to PANI, a partial bond between H of AA and N of PANI appears which increases the inter-chain distance in PANI and the electron hopping mechanism among adjacent chains is disturbed [34]. Consequently, AA is oxidized into dehydroascorbic acid and emeraldine salt is changed into pernagraniline which can again be reduced at moderate electrode potential [25] (Scheme 3).

### 3.7. pH and Scan Rate Effect on AA oxidation

**Figure 6:**
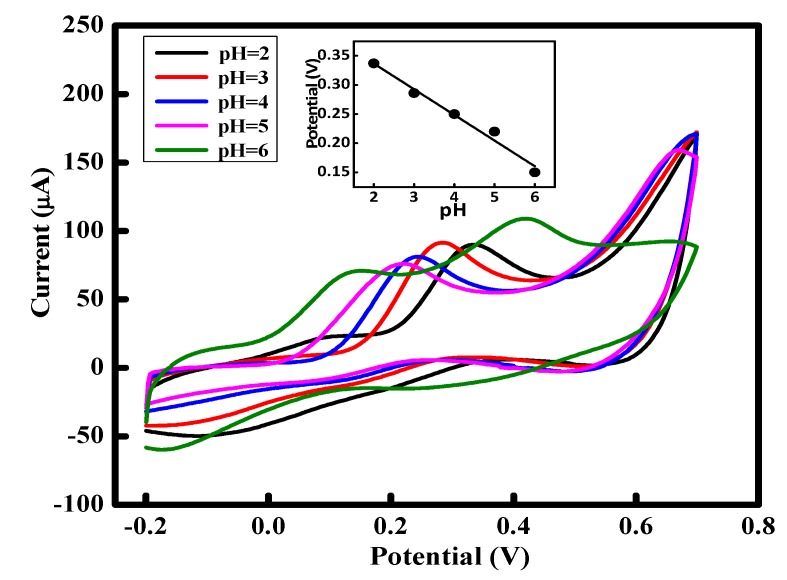
pH effect on the oxidative-current peak of the PANI electrode in AA solution (4mM). at 50 mVs^−1^.

Figure 6 shows the pH influence on the AA oxidation in phosphate buffer solution. The anodic peak of AA is high at low pH and decreases gradually as the pH is increased. The E_pa_ = 0.33 V of AA shift towards negative potential when the pH of PBS increases from 2 to 6. The regression equation of the linear plot between the pH of the electrolyte and peak potential E_p_ *= 424.6 – 44 mVpH* (R^2^ = 0.9733) indicates that AA oxidation is a pH dependent reaction, involving two electrons and one proton [43]. PANI shows high electrical conductivity in acidic solutions which decreases with the increasing pH. Hence, the electro-catalytic oxidation of AA on PANI modified electrode shows an inverse relation with pH of the PBS and the second anodic peak of PANI modified electrode is observed at 0.42V due to is shifted to negative potentials (0.42 V) in high pH PBS (pH = 6). It is also known that AA dissociation occurs into two steps with 4.1 and 11.8 pKa values, respectively. Hence, the reactions of oxidizable species HA^-^ (ionized ascorbate) in high pH solutions are given as under:

HA^-^ → [HA] + ē (2)

HA^-^ + [HA] → A + AA + ē. (3)

The [HA] radical formed in the first step, further oxidized into dehydroascorbic acid (A) while AA is again generated at electrode/electrolyte interface [44].

**Figure 7:**
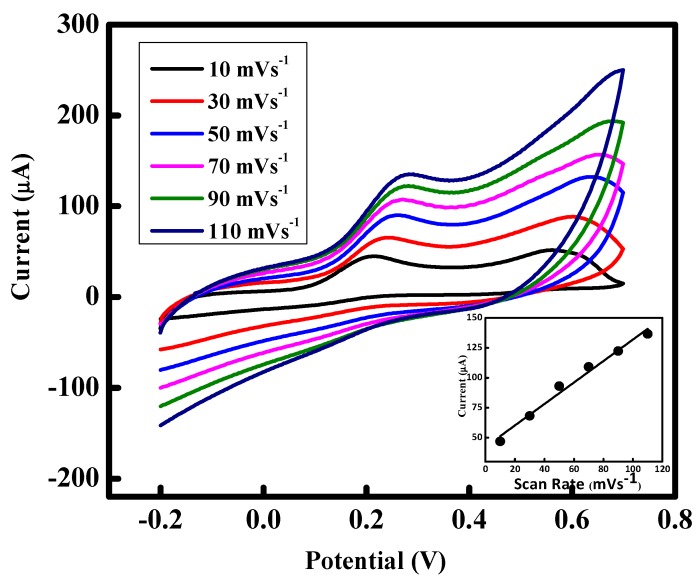
Cyclic voltammograms of the PANI modified electrode, in a solution of 4 mM AA in 0.1 M (pH = 4) phosphate buffer solution (PBS), at different scan rates (10 to 110 mV/s).

The scan rate effect on the anodic current peak of AA was also investigated as depicted in Figure 7 which shows the enhancement in the current peak when the scan rate rises. The anodic-current peak linearly correlates to the υ with regression equation *Ipa (µA) = 42.29 + 0.897*x (R^2^ = 0.980), indicating that the oxidative-process of AA on the modified PANI electrode is electrochemically surface controlled. No well-defined reduction peaks are observed, suggesting that the process is quasi-reversible [45]. 

### 3.8. Effect of AA Concentration on Voltammetric Response of the PANI Modified Electrode 

**Figure 8:**
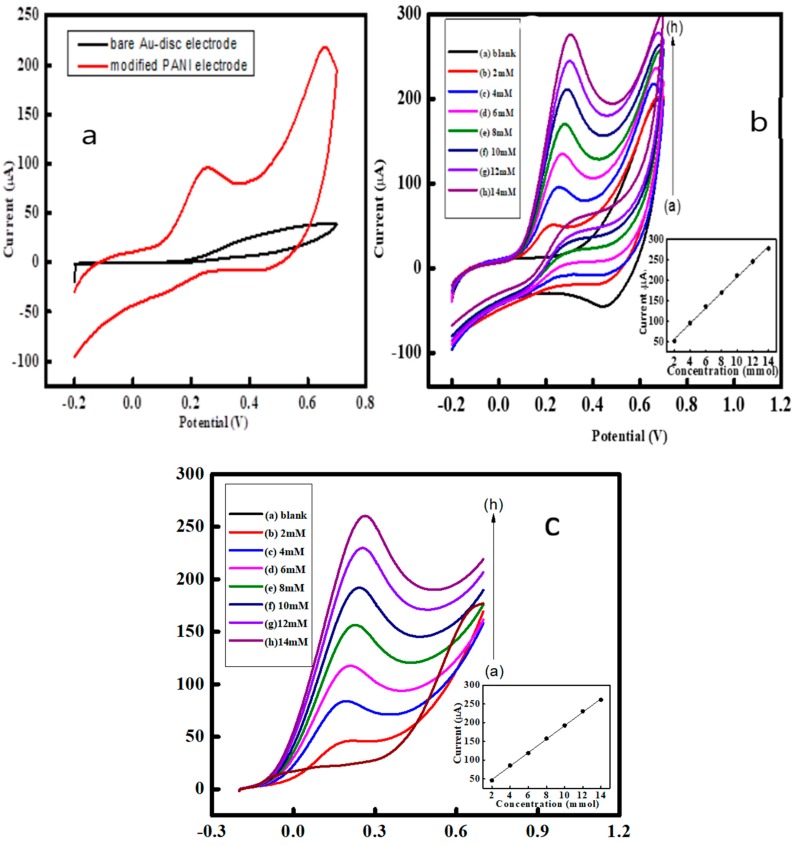
(**a**) Electro-chemical response of bare Au disc and PANI modified electrode towards 4 mM AA in 0.1 M PBS (pH = 4) at scan rate of 50 mV s^−1^. (**b**) Cyclic voltammograms of PANI modified electrode recorded in the presence of various AA concentrations (2–14 mM) in 0.1 M PBS (pH = 4) at scan rate of 50 mV s^−1^. (**c**) Linear sweep voltammetry of the PANI modified electrode in the presence of various AA concentrations (2–14 mM in 0.1 M PBS (pH = 4)) at scan rate of 50 mV s^−1^.

The electrochemical behavior of bare Au disc and PANI modified electrode towards 4 mM AA in 0.1 M PBS (pH = 4) was investigated by recording cyclic voltammograms (CVs) in the potential range of −0.2-0.7 V at 50 mVs^−1^ (Figure 8a). The PANI modified electrode shows a high response towards AA oxidation compared to the bare electrode. It is also evident that oxidation of AA occurs at around 0.26 V on the PANI modified electrode while on the bare gold electrode this value is around 0.4 V. This means the oxidation over potential of AA is decreased by almost 140 mV on the PANI modified electrode as compare to its oxidation potential on the bare gold electrode. 

Figure 8b displays the CVs of the PANI modified electrode recorded for AA solutions of varying concentrations (2-14 mM in 0.1 M PBS (pH = 4)). Anodic peak current increases with increase in AA concentration. This suggests that effective surface area of the electrode is sufficient enough to cope with the increasing analyte concentration and provide sites for its oxidation. The correlation linear equation of the curve shown in the inset of Figure 8b is *I*_pa_*(µA) = 18.8*x *+19.36* (R^2^ = 0.997).

The AA sensing was also studied via linear sweep voltammetry (LSV) at 50 mVs^−1^ as presented in Figure 8c. The observed Epa of AA from LSV was 0.259 V which is approximately the same as found in CV. Again the oxidative-current peak increases with an increase in AA concentration. As discussed in the previous section, the semi-oxidized PANI oxidized AA into dehydroascorbic acid due to electron transfer, indicated by the anodic current peak.

### 3.9. Chronoamperometry

**Figure 9:**
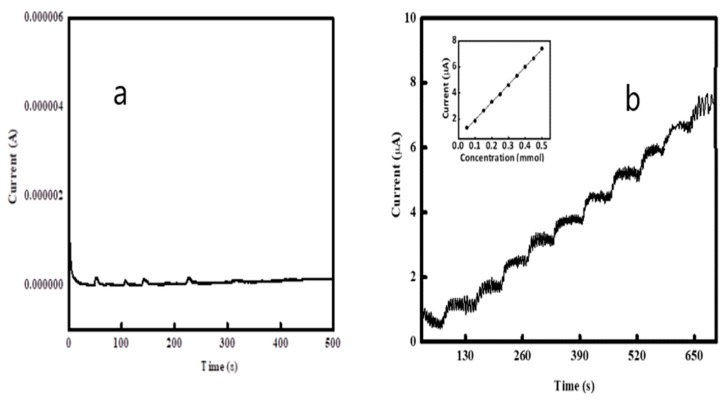
Chronoamperometry of (**a**) a bare Au-disc and (**b**) a PANI modified electrode recorded upon sequential addition of AA (0.05 mM) in 0.1 PBS (pH=4) at a constant voltage of 0.24 V. Inset: dependence of current on AA concentration.

The current response of bare gold (Figure 9a) and PANI modified electrode (Figure 9b) with respect to time was examined when AA (0.05 mM) was successively added to 0.1 M PBS under moderate stirring at an optimized potential of 0.24 V. The response of the bare gold electrode is very low compared to that of the PANI modified electrode. A simple electron transfer process over the surface of the PANI electrode is represented by a steady state plateau which was obtained within 14 s after AA addition, indicating a sensitive response of the modified electrode towards AA oxidation. The steady-state current is linearly correlated to the AA concentration in the range of 0.05 mM to 0.5 mM. The chronoampermetry of PANI is shown in Figure 9b which gives a good calibration curve i.e. *I*_pa_ (µA) = 13.48x + 0.599 (R^2^ = 0.997). The LOD of the electrode was evaluated with the help of following formula

LOD = 3.3 S/b(4)

where “*S*” is the standard deviation while “*b*” is the calibration plot slope.

**Figure 10:**
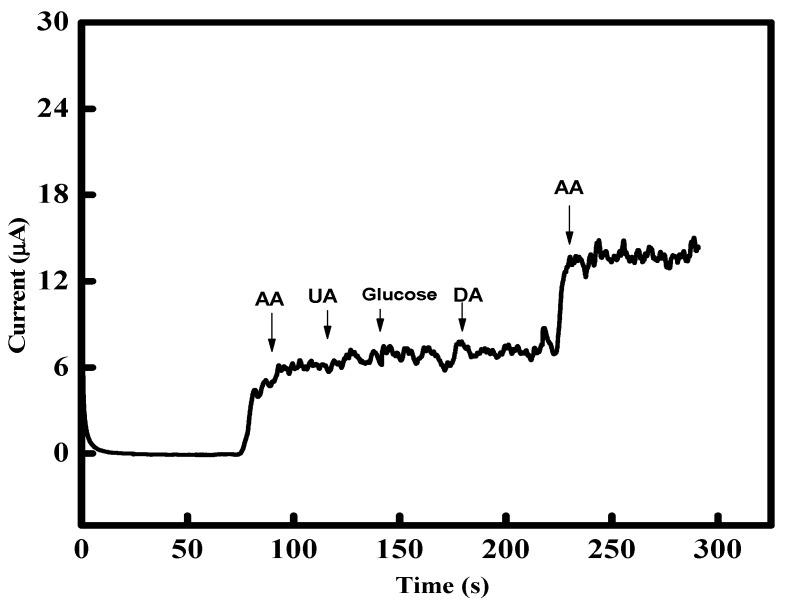
Chronoamperometric response of a PANI electrode to successive addition of 0.3 mM AA, 0.3 mM dopamine (DA), 0.3 mM uric acid (UA), 0.3 mM glucose in 0.1 M PBS at 0.24 V applied potential.

Along with AA many interfering species are also present in the reported usual physiological sample which creates problems during the selective detection of AA. The modified PANI electrode selectivity was investigated by monitoring chronoamperometric responses when AA along with other interfering agents such as dopamine (DA), uric acid (UA) and glucose were added in 0.1 M PBS. Figure 10 shows that the PANI electrode generates significant signals during 0.3 mM AA addition while negligible current was produced when the same amount of interfering species were added. This means that the present PANI based sensor is highly selective towards oxidation of AA. A comparison of the obtained results with already published data by others is provided in Table 1. 

### 3.10. Reproducibility of AA Sensor

**Figure 11:**
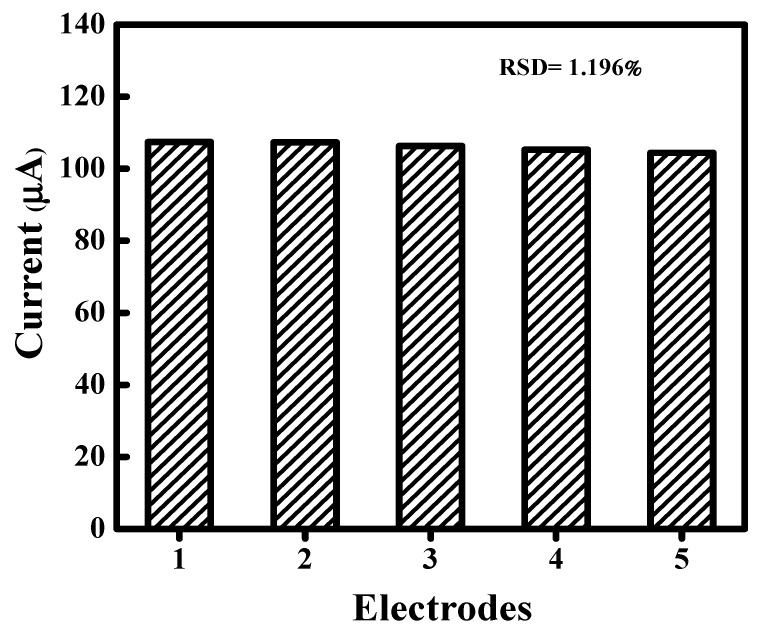
Plot of the current peak of five electrodes modified with PANI.

Figure 11 depicts the reproducibility of the PANI electrode for detection of AA while the relevant values of peak current, standard deviation (SD) and relative standard deviation (RSD) are cited in Table 2. Five PANI electrodes were prepared and their CVs were measured in 0.1 M PBS containing 4 mM AA in order to determine the PANI electrode reproducibility for AA detection. The low RSD value (1.1964%) indicates that the PANI electrode is highly reproducible for AA sensing.

## 4. Conclusions

Electrode modification with conductive PANI via electrochemical polymerization in the presence of DBSA and sulfuric acid can be effectively utilized for selective and catalytic oxidation of ascorbic acid. The catalytic oxidation is attributed to the immense enhancement in the active surface area of the electrode through the PANI coating. The influence of PBS pH and different scan rates on analyte current response was determined in order to understand the optimized conditions for better AA detection. The electrode preparation process is simple and low cost while electrocatalytic behavior of the sensor can possibly pave the way for the fabrication of a reliable AA sensor. The sensor exhibits enhanced properties in terms of detection limit, response time, electrode fabrication and reproducibility in comparison with the other reported AA sensors.

## Figures and Tables

**Table 1:** Comparison of synthesized PANI electrode performance with previously reported data.

Electrode	Linearity (mM)	LOD/(μmol·L^–1^)	Reference
**Cu_4_(OH)_6_SO_4_ Nanorods**	0.017–6	6.4	[46]
**Fe_3_O_4_/r-GO/GC**	0.3–7.2	20	[47]
**Poly(DB71)/GC**	0.001–2	1	[48]
**MWNTs/MGF/GC**	0.1–6	18.28	[49]
**ZnO–GO/GC**	0.005–2	1.2	[50]
**(DBSA)-doped nanoPANI/SPCPE**	0.5–8	8.3	[24]
**PANI-FSA/PGE**	0.001–0.1	1	[51]
**DBSA and H_2_SO_4_ doped PANI / Au-disc**	0.05–0.5	0.0267	This work

GO: graphene oxide, GC: glassy carbon, DB71: direct blue 71, MWNT: multiwall nano tubes, MGF: mesocellular graphene, SPE: screen ptinted carbon paste electrode, FSA/PGE: ferrocene sulfonic acid /pencil graphene oxide electrode.

**Table 2:** Reproducibility of the PANI electrode for sensing AA.

Electrode	1	2	3	4	5	Mean	SD	RSD (%)
**Peak Current (µA)**	107.4	107.3	106.3	105.3	104.4	106.12	1.27	1.1964
